# CANOPY-N: A Phase 2 Study of Canakinumab or Pembrolizumab, Alone or in Combination, as Neoadjuvant Therapy in Patients With Resectable, Stage IB–IIIA NSCLC

**DOI:** 10.1016/j.jtocrr.2025.100859

**Published:** 2025-06-13

**Authors:** Jay M. Lee, Jean-Louis Pujol, Jun Zhang, Oleg Leonov, Masahiro Tsuboi, Edward S. Kim, Calvin Ng, Nicolas Moreno-Mata, Amy Cummings, Ilhan Hacibekiroglu, Abidin Sehitogullari, Nirmal Veeramachaneni, Cathy Spillane, Jiawei Duan, Claudia Bossen, Alexander Savchenko, Chiara Lobetti-Bodoni, Tony Mok, Pilar Garrido

**Affiliations:** a UCLA Health, Los Angeles, California; b Centre Hospitalier Universitaire de Montpellier, Montpellier, France; c University of Kansas Medical Center, Kansas City, Kansas; d Clinical Oncological Dispensary, Omsk, Russia; e National Cancer Center Hospital East, Kashiwa, Japan; f City of Hope National Medical Center, Irvine, California; g Chinese University of Hong Kong, Hong Kong, People's Republic of China; h Hospital Universitario Ramón y Cajal, Madrid, Spain; i Sakarya University, Sakarya, Turkey; j Saint Louis University School of Medicine, Saint Louis, Missouri; k Novartis Pharmaceuticals Corporation, Dublin, Ireland; l Novartis Pharma AG, Basel, Switzerland; m Novartis Pharmaceuticals Corporation, Cambridge, Massachusetts

**Keywords:** NSCLC, Neoadjuvant treatment, Monoclonal antibodies

## Abstract

**Introduction:**

Canakinumab is a human monoclonal anti–interleukin-1β antibody with the potential to enhance the activity of programmed death-ligand 1 inhibitors by inhibiting protumor inflammation.

**Methods:**

CANOPY-N was a randomized, phase 2 study to evaluate safety and efficacy of neoadjuvant canakinumab (200 mg subcutaneous once every three weeks) and pembrolizumab (200 mg intravenous once every three weeks), either in combination or alone, in patients with early-stage (stage Ib–IIIa) NSCLC. The primary end point was major pathologic response (MPR) rates (≤10% of residual tumor cells) by central pathology review in the arms containing canakinumab. Secondary end points included overall response rates, safety, pharmacokinetics, surgical feasibility rates, and MPR rate in the pembrolizumab arm. The impact of treatment on surgical outcomes was assessed as an exploratory outcome.

**Results:**

In total, 88 patients were enrolled: 35 to the canakinumab arm, 35 to the canakinumab + pembrolizumab arm, and 18 to the pembrolizumab arm. One patient (2.9%) in the canakinumab arm (95% confidence interval [CI]: 0.07–14.92), six patients (17.1%) in the canakinumab + pembrolizumab arm (95% CI: 6.56–33.65), and three patients (16.7%) in the pembrolizumab arm (95% CI: 3.58–41.42) achieved MPR. No unexpected safety signals were observed. Of the 84 patients (95.5%) who underwent operation, the prespecified 6-week window was achieved for 72 patients (85.7%).

**Conclusions:**

Neoadjuvant treatment with canakinumab alone or combined with pembrolizumab did not improve MPR rates compared with pembrolizumab alone. No unexpected safety signals were observed and canakinumab did not adversely affect surgical outcomes. Intraoperative perihilar or perilobular fibrosis after neoadjuvant immunotherapy was rare.

## Introduction

Lung cancer is the most frequent cause of cancer-related deaths worldwide,^1^ with NSCLC accounting for approximately 85% of all lung cancers.^2^ Surgical resection is the treatment cornerstone for patients with early-stage NSCLC,^3^ with platinum-based chemotherapy (CT) typically administered as neoadjuvant or adjuvant treatment, which confers a statistically significant, but modest, survival benefit.^4^^,^^5^ Recently, the addition of immune checkpoint inhibitors (ICIs) to (neo)adjuvant platinum-based CT has been found to yield an event-free survival benefit for patients with early-stage NSCLC.^6^, ^7^, ^8^, ^9^ In addition, preoperative ICI plus CT in perioperative trials has shown event-free survival^10^, ^11^, ^12^ and overall survival (OS)^13^ benefit. However, despite recent advances in early-stage NSCLC management, the long-term prognosis for patients remains poor, with disease recurrence occurring frequently. Therefore, alternative and more effective treatment options are needed.

Interleukin-1β (IL-1β) facilitates tumor growth and promotes invasiveness and metastases in preclinical models of NSCLC, and high IL-1β expression has been associated with a poor prognosis among patients with early-stage NSCLC.^14^, ^15^, ^16^ Canakinumab is a high-affinity, human monoclonal antibody that selectively targets IL-1β and blocks its interaction with its receptor, and has been shown to potentiate the antitumor activity of ICIs in NSCLC preclinical models.^17^ Furthermore, in a post hoc analysis of the phase 3 Canakinumab Anti-inflammatory Thrombosis Outcomes Study (CANTOS; NCT01327846) that investigated canakinumab for the secondary prevention of major cardiovascular events, significant reductions in both lung cancer incidence and mortality were observed in patients treated with canakinumab in a dose-dependent manner, building the rationale to explore IL-1β as a therapeutic target in patients with NSCLC.^18^

Here we present the findings from CANOPY-N, a randomized, phase 2 study aimed at determining the efficacy and safety of canakinumab and pembrolizumab, either in combination or alone, as neoadjuvant treatment in patients with early-stage resectable NSCLC.^19^

## Methods

### Study Design

**Figure 1:**
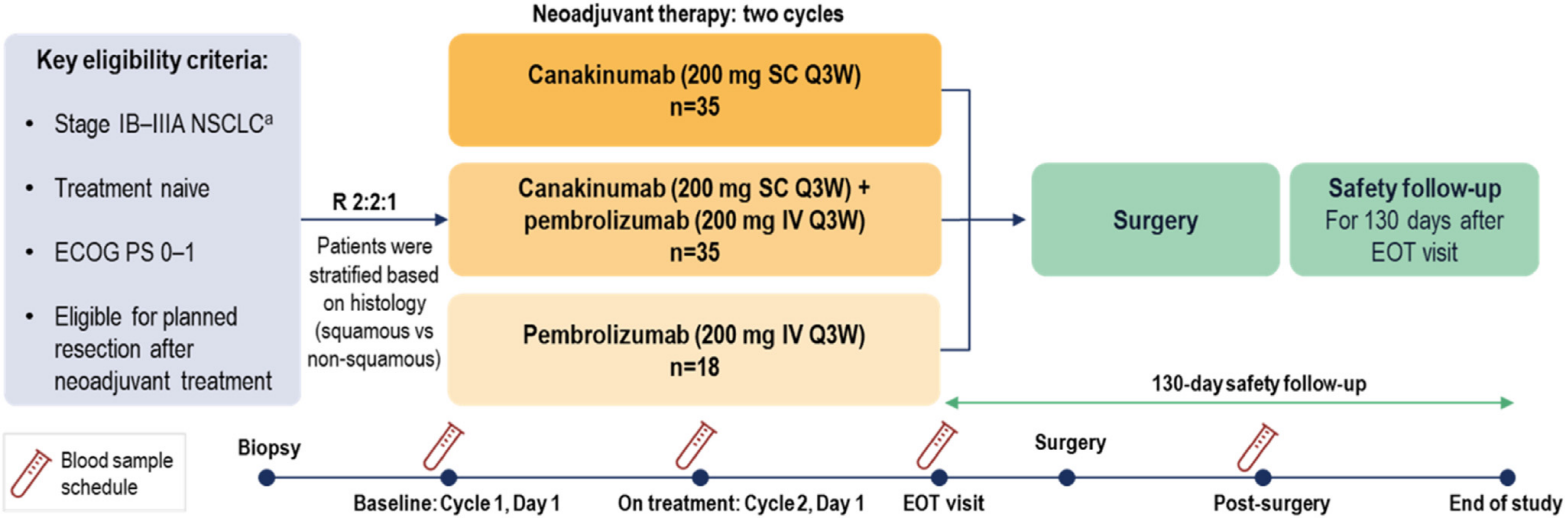
CANOPY-N study design. ^a^Stages were defined using the AJCC/UICC Staging Manual, version 8. AJCC, American Joint Committee on Cancer; ECOG, Eastern Cooperative Oncology Group; EOT, end of treatment; IV, intravenously; MPR, major pathologic response; PS, performance status; Q3W, once every three weeks; R, randomized; SC, subcutaneously; UICC, Union for International Cancer Control.

The CANOPY-N trial (NCT03968419) was a phase 2, randomized, open-label study evaluating efficacy and safety of canakinumab or pembrolizumab, either alone or in combination, compared with pembrolizumab alone as neoadjuvant treatment in patients with stage IB–IIIA NSCLC (Fig. 1). The study recruited from 29 sites across 12 countries and approximately 110 patients were planned to be randomized in a 2:2:1 ratio to one of the treatment arms: canakinumab 200 mg subcutaneous once every three weeks (Q3W), canakinumab 200 mg subcutaneous Q3W plus pembrolizumab 200 mg intravenous Q3W, and pembrolizumab 200 mg intravenous Q3W. Randomization was stratified by histology (squamous versus nonsquamous). With 44 patients randomized to the canakinumab arm (canakinumab + pembrolizumab arm), the probability of erroneously declaring proof of efficacy is at most 4.4% (2.1%) when the true major pathologic response (MPR) rate is ≤20% (≤30%) whereas the probability of declaring proof of efficacy is at least 89.8% (92.2%) for MPR rate of greater than or equal to 40% (≥55%). Recruitment was interrupted earlier than predicted per the protocol.

### Patient Population

Patients were considered eligible if they were greater than or equal to 18 years, had stage IB–IIIA NSCLC (per the American Joint Committee on Cancer Staging Manual, version 8) regardless of programmed death-ligand 1 (PD-L1) status, were deemed suitable for primary resection four to six weeks from the first study treatment administration by a multidisciplinary team including a surgeon, had an Eastern Cooperative Oncology Group performance status of 0 to 1, and adequate organ function.

Key exclusion criteria were history of autoimmune disease, unresectable or metastatic disease, and previous systemic anticancer therapy within three years before screening. All patients were required to have had brain imaging before enrollment to exclude brain metastasis. Testing for actionable mutations was not required per protocol.

### Treatment

Patients were treated for a maximum duration of six weeks (two cycles), and for both canakinumab and pembrolizumab, dose modifications were not allowed. Canakinumab dose interruptions for a maximum of one week were allowed, and pembrolizumab dose interruptions followed locally approved labels and local clinical practice. Discontinuation from the study could occur under the following circumstances: patient or physician’s decision, pregnancy, disease progression, or unacceptable toxicity. The operation was performed as per local guidelines and clinical practice, approximately four to six weeks after the first administration of study treatment (day 1). Adjuvant treatment was not mandated per protocol and could be administered at the investigator’s discretion.

### Study Objectives and End Points

The primary objective was to assess the antitumor activity of canakinumab alone or combined with pembrolizumab compared with pembrolizumab alone, and the primary end point was MPR, defined as the presence of ≤10% of residual viable tumor cells in the surgical specimen, per central pathology review. Patients who started new antineoplastic therapies before operation, who could not be submitted to operation, or whose MPR assessment was unevaluable were considered to have a non-MPR.

Key secondary objectives included safety and tolerability, MPR rates by local review, overall response rate based on local investigator assessment per Response Evaluation Criteria in Solid Tumors version 1.1 (RECIST v1.1), pharmacokinetics (PK), and surgical feasibility rates.

Key exploratory end points included evaluating the impact of the study treatment on surgical outcomes.

### Assessments

Efficacy was evaluated by means of the primary end point of MPR, defined as ≤10% of residual viable tumor cells in primary tumor tissue, which was assessed locally and centrally.

MPR was established to assess the amount of residual viable tumor cells present in the original tumor bed using hematoxylin and eosin (H&E)-stained slides. The methodology was validated at the partner Clinical Laboratory Improvement Amendments laboratory following the International Association for the Study of Lung Cancer–published recommendations for the pathologic assessment of lung cancer resection specimens after neoadjuvant therapy.^20^

The size of the tumor bed was determined at the extent of sampling and histologic H&E assessment. At least one tissue section per diameter of the tumor bed after representative H&E staining was collected when the tumor bed was larger than 3 cm. Tumor beds measuring below 3 cm at the widest diameter were entirely sampled for complete histologic sectioning and scoring. Histologic sections at the periphery of the tumor bed included 1 cm of the surrounding non-neoplastic lung tissue to define the edge of the tumor. The percentage of residual viable tumor cells was calculated as the estimated size of viable tumor component divided by the size of the tumor bed. In addition, percentage of stromal (including inflammation or fibrosis) and necrotic components were determined and reported. The total tumor bed composed of each histologic component equaled 100%.

PD-L1 expression was assessed using the IHC 22C3 PharmDx assay. The fraction of viable tumor cells (tumor proportion score; calculated as a percentage) that expressed PD-L1 was scored.^21^

In addition, response rates were assessed centrally on both the primary tumor and the lymph node. Radiology tumor assessments (positron emission tomography scan and computed tomography or magnetic resonance imaging) were performed by a local investigator based on RECIST v1.1 at screening (baseline) and before operation.

Adverse events (AEs) were summarized by system organ class and preferred term using Medical Dictionary for Regulatory Activities coding by treatment arm during the on-treatment period. Grades were assessed per Common Terminology Criteria for Adverse Events version 5.0.

### Statistical Analysis

The full analysis set (FAS) comprised all patients randomized in the study. According to the intent-to-treat principle, patients were analyzed according to the treatment they had been assigned to during the randomization procedure. The safety set included all patients who received at least one dose of study treatment. The PK Analysis Set comprised all patients who received at least one dose of study drug and had at least one evaluable PK sample, defined for canakinumab and pembrolizumab separately.

The statistical assumption when the study was conceived was that platinum-based CT, the standard neoadjuvant treatment for patients with NSCLC, can yield an MPR in up to 20% of the cases.^22^ For canakinumab alone, a 10% absolute improvement in the MPR to 30% would be considered a clinically meaningful minimum improvement in this population. Therefore, proof of efficacy in the canakinumab alone arm would be declared if both of the following conditions were met: the mean of the posterior distribution of MPR was at least 30%, and there was at least 90% probability that the MPR rate was greater than or equal to 20%.

For the combination of canakinumab and pembrolizumab arm, a 25% absolute improvement in the MPR to 45% would be considered a clinically meaningful minimum improvement in this population. Therefore, proof of efficacy in the combination treatment arm was declared if both of the following conditions were met: the mean of the posterior distribution of MPR was at least 45%, and there was at least 90% probability that the MPR rate was greater than or equal to 30%.

The posterior distribution of MPR rate was derived from the previous distribution and all available data from the patients in the FAS. The posterior mean of MPR rate was calculated, and the MPR rate with its corresponding two-sided exact binomial 95% confidence interval (CI) was estimated in both the canakinumab and canakinumab + pembrolizumab arms.^23^

The overall observation period for safety analysis was divided into three mutually exclusive segments:

1. Pretreatment period: from the day of informed consent to the day before the first dose of study treatment.
2. On-treatment period: from the day of the first dose of study treatment to 130 days after the last dose of study treatment.
3. Posttreatment period: starting at day 131 after the last dose of any component of the study treatment.

### Statistical Analysis: Surgical Exploratory End Points

The safety information collected at the time of operation was summarized and listed using the FAS. Time from screening to operation, duration of the surgical procedure (from incision to closure), and estimated blood loss (mL) were summarized using descriptive statistics by treatment arm. For the rest of the variables, frequency and percentages of patients in each category were provided by treatment arm. Analysis was performed for surgical end points and covered preoperative, intraoperative, and postoperative end points, including preoperative and postoperative attrition rates. Neoadjuvant end points included overall tumor downstaging and nodal downstaging. Intraoperative end points included resection completeness, estimated blood loss, intraoperative fibrosis (mediastinal and peripheral), and rates of chest tube removal within five days. Postoperative end points included immune-mediated AEs (imAEs) and 30- and 90-day postoperative mortality.

### Ethical Oversight

The protocol and all amendments were approved by the independent ethics committee or institutional review board at each participating study site. The study was conducted in accordance with the principles of the Declaration of Helsinki and the International Conference on Harmonization Good Clinical Practice guidelines. All patients provided a written informed consent before randomization.

## Results

### Patient Demographics and Disposition

As of the data cutoff, 88 patients were enrolled in the study: 35 to the canakinumab arm, 35 to the canakinumab + pembrolizumab arm, and 18 to the pembrolizumab arm. Patient characteristics were well balanced among treatment arms; 59% of patients were male, 55% had adenocarcinoma histology, 61% had an Eastern Cooperative Oncology Group performance status of 0, and 86.4% were former or current smokers (Table 1).

**Table 1:** Patient Demographics

Demographic Variable	Canakinumab (n = 35)	Canakinumab + Pembrolizumab (n = 35)	Pembrolizumab (n = 18)	All Patients (N = 88)
Median age, y (IQR)	66.0 (56.0–75.0)	68.0 (62.0–72.0)	67.0 (61.0–70.0)	67.0 (61.0–71.5)
Sex, n (%)				
Male	22 (62.9)	21 (60.0)	9 (50.0)	52 (59.1)
Female	13 (37.1)	14 (40.0)	9 (50.0)	36 (40.9)
ECOG performance status, n (%)				
0	22 (62.9)	19 (54.3)	13 (72.2)	54 (61.4)
1	13 (37.1)	16 (45.7)	5 (27.8)	34 (38.6)
Smoking history, n (%)				
Current smoker or history of smoking	32 (91.4)	29 (82.9)	15 (83.3)	76 (86.4)
Never smoked	3 (8.6)	6 (17.1)	3 (16.7)	12 (13.6)
Stage at time of study entry				
IB	8 (22.9)	10 (28.6)	5 (27.8)	23 (26.1)
IIA	8 (22.9)	4 (11.4)	4 (22.2)	16 (18.2)
IIB	13 (37.1)	17 (48.6)	7 (38.9)	37 (42.0)
IIIA	6 (17.1)	4 (11.4)	2 (11.1)	12 (13.6)
Histology/cytology				
Adenocarcinoma	18 (51.4)	19 (54.3)	11 (61.1)	48 (54.5)
Squamous cell carcinoma	15 (42.9)	14 (40.0)	7 (38.9)	36 (40.9)
Other	2 (5.7)	2 (5.7)	0	4 (4.5)
Biomarker, n (%)				
PD-L1 <1%	12 (34.3)	14 (40.0)	7 (38.9)	33 (37.5)
PD-L1 1%–49%	15 (42.9)	13 (37.1)	6 (33.3)	34 (38.6)
PD-L1 ≥50%	6 (17.1)	7 (20.0)	3 (16.7)	16 (18.2)
Missing	2 (5.7)	1 (2.9)	2 (11.1)	5 (5.7)

ECOG, Eastern Cooperative Oncology Group; IQR, interquartile range; PD-L1, programmed death-ligand 1.

In total, 87 of the 88 participants completed the planned two cycles of neoadjuvant treatment. One patient in the pembrolizumab arm discontinued treatment after the first cycle, and one patient in the canakinumab + pembrolizumab arm discontinued pembrolizumab after the first cycle, but continued canakinumab treatment.

The median duration of exposure to canakinumab was 6.0 weeks in both the canakinumab (range, 5.6–6.3 weeks) and the canakinumab + pembrolizumab arms (range, 4.7–7.0 weeks).

The median duration of exposure to pembrolizumab was 6.0 weeks in both the pembrolizumab (range, 3.0–6.3 weeks) and the canakinumab + pembrolizumab arms (range, 3.0–7.0 weeks).

### Efficacy

Overall, one patient (2.9%) in the canakinumab arm (95% CI: 0.07–14.92), six patients (17.1%) in the canakinumab + pembrolizumab arm (95% CI: 6.56–33.65), and three patients (16.7%) in the pembrolizumab arm (95% CI: 3.58–41.42) achieved an MPR per central review (analysis of the primary end point are presented in Supplementary Table S1).

No patients in the canakinumab arm (95% CI: 0.00–10.00), 20.0% of the patients in the canakinumab + pembrolizumab arm (95% CI: 8.44–36.94), and 22.2% of patients in the pembrolizumab arm (95% CI: 6.41–47.64) achieved an MPR per local assessment. Owing to the small number of patients achieving MPR, no subgroup analyses were performed.

No patients in the canakinumab arm achieved the best overall response of complete response or partial response per RECIST v1.1. Partial responses were achieved by 8.6% (3/35) and 11.1% of patients (2/18) in the canakinumab + pembrolizumab arm and the pembrolizumab arm, respectively (Supplementary Table S2).

### Safety

Overall, 78 patients (88.6%) experienced at least one AE (Table 2) and no unexpected safety signals were observed with canakinumab alone or in combination.

**Table 2:** Overview of Adverse Events

Adverse Event	Canakinumab (n = 35)			Canakinumab + Pembrolizumab (n = 35)			Pembrolizumab (n = 18)		
	All grades, n (%)	Grade 3/4, n (%)	Grade 5, n (%)	All grades, n (%)	Grade 3/4, n (%)	Grade 5, n (%)	All grades, n (%)	Grade 3/4, n (%)	Grade 5, n (%)
All deaths	3 (8.6)	0	3 (8.6)	2 (5.7)	0	1 (2.9)	1 (5.6)	0	1 (5.6)
On-treatment deaths	3 (8.6)	0	3 (8.6)	2 (5.7)	0	1 (2.9)	1 (5.6)	0	1 (5.6)
Adverse events	31 (88.6)	10 (28.6)	3 (8.6)	32 (91.4)	9 (25.7)	1 (2.9)	15 (83.3)	3 (16.7)	1 (5.6)
Treatment related	13 (37.1)	0	0	13 (37.1)	4 (11.4)	0	9 (50.0)	2 (11.1)	0
SAEs	10 (28.6)	6 (17.1)	3 (8.6)	9 (25.7)	4 (11.4)	1 (2.9)	4 (22.2)	3 (16.7)	1 (5.6)
Treatment related	0	0	0	2 (5.7)	2 (5.7)	0	1 (5.6)	1 (5.6)	0
Fatal SAEs	3 (8.6)	0	3 (8.6)	1 (2.9)	0	1 (2.9)	1 (5.6)	0	1 (5.6)
Treatment related	0	0	0	0	0	0	0	0	0
AEs leading to any drug discontinuation	0	0	0	1 (2.9)	1 (2.9)	0	1 (5.6)	1 (5.6)	0
Treatment related	0	0	0	1 (2.9)	1 (2.9)	0	0	0	0
AEs leading to canakinumab discontinuation	0	0	0	0	0	0	0	0	0
AEs leading to any dose interruption	0	0	0	1 (2.9)	1 (2.9)	0	0	0	0
AEs requiring additional therapy	19 (54.3)	7 (20.0)	2 (5.7)	25 (71.4)	7 (20.0)	1 (2.9)	11 (61.1)	4 (22.2)	0
Treatment related	1 (2.9)	0	0	13 (37.1)	4 (11.4)	0	4 (22.2)	2 (11.1)	0
All imAEs	0	0	0	9 (25.7)	1 (2.9)	0	3 (16.7)	1 (5.6)	0
imAEs requiring additional therapy:	0	0	0	8 (22.9)	1 (2.9)	0	2 (11.1)	1 (5.6)	0
Steroids	0	0	0	3 (8.6)	1 (2.9)	0	0	0	0
Hormonal therapy	0	0	0	5 (14.3)	0	0	2 (11.1)	1 (5.6)	0

AE, adverse event; imAE, immune-mediated AE; SAE, serious AE.

Treatment-emergent AEs of any grade were comparable among arms and were reported in 31 (88.6%), 32 (91.4 %), and 15 patients (83.3%) in the canakinumab, canakinumab + pembrolizumab, and pembrolizumab treatment arms, respectively. All AEs reported in greater than or equal to 10% of patients are presented in Supplementary Table S3.

Treatment-related AEs (TRAEs) of any grade were reported in 37.1% of patients in both the canakinumab arm and the canakinumab + pembrolizumab arm and 50% of patients in the pembrolizumab arm. Grade ≥3 TRAEs were reported in no patients in the canakinumab arm, four patients (11.4%) in the canakinumab + pembrolizumab arm, and two patients (11.1%) in the pembrolizumab arm. No grade 5 TRAEs were reported in any arm (Table 3).

**Table 3:** Treatment-Related AEs by Preferred Term and Grade (in ≥5% of Patients, All Grades, Any Treatment Arm)

Adverse Event	Canakinumab (n = 35)			Canakinumab + Pembrolizumab (n = 35)			Pembrolizumab (n = 18)		
	All grades, n (%)	Grade 3/4, n (%)	Grade 5, n (%)	All grades, n (%)	Grade 3/4, n (%)	Grade 5, n (%)	All grades, n (%)	Grade 3/4, n (%)	Grade 5, n (%)
Number of patients with ≥1 TRAE	13 (37.1)	0	0	13 (37.1)	4 (11.4)	0	9 (50.0)	2 (11.1)	0
Bilirubin conjugated increased	4 (11.4)	0	0	0	0	0	1 (5.6)	0	0
Blood bilirubin increased	2 (5.7)	0	0	0	0	0	0	0	0
Decreased appetite	2 (5.7)	0	0	0	0	0	0	0	0
Fatigue	2 (5.7)	0	0	1 (2.9)	0	0	2 (11.1)	0	0
Lymphocyte count decreased	2 (5.7)	0	0	0	0	0	0	0	0
Nausea	1 (2.9)	0	0	0	0	0	1 (5.6)	0	0
ALT increased	0	0	0	3 (8.6)	1 (2.9)	0	1 (5.6)	0	0
Amylase increased	0	0	0	1 (2.9)	0	0	1 (5.6)	0	0
AST increased	0	0	0	3 (8.6)	1 (2.9)	0	0	0	0
Dry mouth	0	0	0	1 (2.9)	0	0	1 (5.6)	0	0
Dysgeusia	0	0	0	2 (5.7)	0	0	0	0	0
Hyperglycemia	0	0	0	2 (5.7)	2 (5.7)	0	0	0	0
Hypertension	0	0	0	2 (5.7)	1 (2.9)	0	0	0	0
Hyperthyroidism	0	0	0	4 (11.4)	0	0	1 (5.6)	0	0
Hypophosphatemia	0	0	0	0	0	0	1 (5.6)	1 (5.6)	0
Hypothyroidism	0	0	0	3 (8.6)	0	0	2 (11.1)	1 (5.6)	0
Influenza-like illness	0	0	0	0	0	0	1 (5.6)	0	0
Lipase increased	0	0	0	1 (2.9)	0	0	1 (5.6)	0	0
Micturition urgency	0	0	0	0	0	0	1 (5.6)	0	0
Pruritus	0	0	0	2 (5.7)	0	0	0	0	0
Rash pustular	0	0	0	0	0	0	1 (5.6)	0	0

ALT, alanine aminotransferase; AST, aspartate aminotransferase; TRAE, treatment-related adverse event.

Infections of any grade, reported by system organ class, occurred in 11 patients (31.4%) in the canakinumab arm, of which three (8.6%) were grade 3/4 and one (2.9%) was grade 5; five patients (14.3%) in the canakinumab + pembrolizumab arm, of which two (5.7%) were grade 3/4 with no grade 5 cases; and seven (38.9%) in the pembrolizumab arm, of which one (5.6%) was grade 3/4 and one (5.6%) was grade 5. Neutropenia was reported in seven patients (20%) in the canakinumab arm, of which one (2.9%) was grade 3/4 with no grade 5 reported. In the pembrolizumab arm, one patient (2.6%) reported neutropenia below grade 3. No neutropenia was reported in the canakinumab + pembrolizumab arm (Supplementary Table S4).

Two patients experienced AEs that led to treatment discontinuation. One patient in the pembrolizumab arm discontinued owing to pleural hemothorax, which was not related to study treatment, but to a biopsy performed at screening. One patient in the canakinumab + pembrolizumab arm discontinued pembrolizumab owing to increased alanine aminotransferase and aspartate aminotransferase, but completed the planned two cycles of canakinumab.

imAEs occurred in nine patients (25.7%) in the canakinumab + pembrolizumab arm and three patients (16.7%) in the pembrolizumab arm. No imAEs occurred in the canakinumab arm. All imAEs are presented in Table 2. A complete list of the imAEs by preferred term is presented in Supplementary Table S5.

Six on-treatment deaths occurred, all in the postoperative period (within 90 days of operation), none of which were considered to be related to study treatment. One death was caused by progressive disease in the canakinumab + pembrolizumab arm. Five of the deaths were caused by AEs not related to the study treatment: pneumonia (n = 1), cardiac failure (n = 1), and postoperative respiratory failure (n = 1) in the canakinumab arm; pulmonary edema (n = 1) in the canakinumab + pembrolizumab arm; and coronavirus disease 2019 infection (n = 1) in the pembrolizumab arm.

### PK

Thirty-four patients (97.1%) in the canakinumab + pembrolizumab arm and 17 patients (94.4%) in the pembrolizumab arm were included in the pembrolizumab PK analysis set.

Canakinumab and pembrolizumab concentrations were similar in both the monotherapy and combination arms. The geometric mean predose serum canakinumab concentration on cycle 2 day 1 was 10.9 μg/mL with interpatient variability of 32.9% for the canakinumab arm. The geometric mean predose serum canakinumab concentration on cycle 2 day 1 was 10.3 μg/mL with interpatient variability of 41.0% for the canakinumab + pembrolizumab arm.

The geometric mean predose serum pembrolizumab concentration on cycle 2 day 1 was 13.7 μg/mL with interpatient variability of 39.9% for the pembrolizumab arm, unchanged from the primary analysis. The geometric mean predose serum concentration on cycle 2 day 1 was 16.0 μg/mL with interpatient variability of 43.4% for the canakinumab + pembrolizumab arm.

In the canakinumab arm, eight patients had a C_trough_ of greater than or equal to Q3. Because only one of eight patients achieved MPR in this subgroup (12.5%), no conclusions could be drawn on the trend of MPR by PK. No relevant trend was observed in the canakinumab + pembrolizumab arm as MPR rate tended to increase with an increase in C_trough_ quartile up to ≥Q2 to <Q3 and then decreased for C_trough_ ≥Q3.

### Surgical End Points

Of the 84 patients (95.5%) who underwent operation, 72 (85.7%) had surgical resection within the prespecified window, at a median time of 2.1, 2.2, and 2.1 weeks from the last dose of study treatment in the canakinumab, canakinumab + pembrolizumab, and pembrolizumab arms, respectively (Supplementary Table S6).

In total, 32 patients (91.4%) in the canakinumab arm, 34 patients (97.1%) in the canakinumab + pembrolizumab arm, and 18 patients (100%) in the pembrolizumab arm underwent operation; three patients did not undergo operation in canakinumab arm owing to radiologically confirmed disease progression and one patient chose not to undergo operation in the canakinumab + pembrolizumab arm.

Complete resection (R0) was performed in 29 patients (90.6%) in the canakinumab arm, 34 patients (100%) in the canakinumab + pembrolizumab arm, and 17 patients (94.4%) in the pembrolizumab arm (Table 4).

**Table 4:** Intraoperative and Postoperative Surgical End Points

Surgical and Clinical End Points	Canakinumab(n = 32)	Canakinumab + Pembrolizumab (n = 34)	Pembrolizumab (n = 18)
Intraoperative			
R0 resection, n (%)	29 (90.6)	34 (100)	17 (94.4)
R1 resection, n (%)	2 (6.3)	0	0
R2 resection, n (%)^a^	1 (3.1)	0	1 (5.6)
Minimally invasive operation, n (%)	14 (43.8)	19 (55.9)	10 (55.6)
Conversion to thoracotomy, n (%)	6 (18.8)	0	2 (11.1)
Median duration of operation, min (range)	180 (38–429) [n = 27]	145 (76–455) [n = 29]	180.5 (75–334) [n = 14]
Intraoperative complications, n (%)^b^	1 (3.1)	1 (2.9)	2 (11.1)
Mean estimated blood loss, mL	259 [n = 19]	228 [n = 22]	71 [n = 11]
Lung cancer location, n (%)			
Central	16 (50.0)	18 (52.9)	5 (27.8)
Peripheral	10 (31.3)	13 (38.2)	7 (38.9)
Other	5 (15.6)	3 (8.8)	6 (33.3)
Not reported	1 (3.1)^c^	0	0
Intraoperative mediastinal fibrosis, n (%)			
Yes	1 (3.1)	1 (2.9)	1 (5.6)
No	18 (56.3)	24 (70.6)	15 (83.3)
Unknown	13 (40.6)	9 (26.5)	2 (11.1)
Intraoperative peripheral fibrosis, n (%)			
Yes	4 (12.5)	3 (8.8)	2 (11.1)
No	16 (50.0)	20 (58.8)	14 (77.8)
Unknown	12 (37.5)	11 (32.4)	2 (11.1)
Perihilar or perilobular fibrosis, n (%)^d^			
Yes	2 (6.3)	2 (5.9)	2 (11.1)
Grade 1	1 (3.1)	1 (2.9)	2 (11.1)
Grade 2	1 (3.1)	0	0
Grade 3	0	1 (2.9)	0
No	17 (53.1)	21 (61.8)	14 (77.8)
Unknown	13 (40.6)	11 (32.4)	2 (11.1)
Chest tube removal >5 d after operation, n (%)	12 (37.5)	8 (23.5)	6 (33.3)
Reason for delayed removal			
Fluid drainage	8 (25.0)	4 (11.8)	3 (16.7)
Bronchopleural fistula/prolonged air leak	4 (12.5)	2 (5.9)	0
Pneumothorax	0	1 (2.9)	0
Hemothorax	0	0	1 (5.6)
Pulmonary embolism	0	1 (2.9)	0
Chest tube management	0	0	1 (5.6)
Serous debris	0	0	1 (5.6)
Pathologic response by central assessment, n (%)	31 (96.9)	34 (100)	17 (94.4)
Reason for no pathologic assessment			
Intraoperative unresectability	1 (3.1)	0	1 (5.6)
MPR rate by central assessment, % (95% CI)	2.9 (0.07–14.92) [n = 35]	17.1 (6.56–33.65) [n = 34]	11.1 (1.38–34.71) [n = 18]
Postoperative			
Postoperative grade ≥3 TRAEs, n (%)	0	1 (2.9)	2 (11.1)
Number of deaths (days from operation), n			
30 d	2	1	0
90 d^e^	3	2	1
Postoperative morbidity (selected), n (%)^f^			
Cardiac failure	1 (3.3) [n = 30]	0	0
Pneumonitis	0	0	0
Pneumonia	1 (3.3) [n = 30]	0	0
Pulmonary edema	0	1 (3.0) [n = 33]	0
Respiratory failure	1 (3.3) [n = 30]	0	0
ARDS	0	0	0
Bronchopleural fistula/prolonged air leak	0	0	0

ARDS, acute respiratory distress syndrome; CAN, canakinumab; CI, confidence interval; MPR, major pathologic response; PEM, pembrolizumab; TRAE, treatment-related adverse event.

a Physician decision of open–close operation owing to demonstration of progressive disease during surgical procedure.

b One patient in each of the CAN and CAN + PEM arms had pulmonary artery injury, and in the PEM arm, one patient had right hemothorax and one patient had vocal cord injury.

c Lesion described as being in the left upper lobe of the lung.

d Gradings are presented in Supplementary Table S7.

e Cumulative deaths from day 0 to 90.

f n represents the patients with at least 90 days of follow-up after operation.

Perihilar or perilobular fibrosis was reported in two patients in each treatment arm, of which one (2.9%) was grade 3 in the canakinumab + pembrolizumab arm and one (3.1%) was grade 2 in the canakinumab arm.

Postoperative grade ≥3 TRAEs were reported in one patient (2.9%) in the canakinumab + pembrolizumab arm (hyperglycemia and hypertension) and two patients (11.1%) in the pembrolizumab arm (hypophosphatemia and hypothyroidism, respectively). No grade ≥3 TRAEs were reported in the canakinumab arm.

## Discussion

CANOPY-N did not meet its primary end point of MPR rates for either of the canakinumab-containing arms. Although the end point was not met, neoadjuvant treatment did not compromise surgical outcomes or lead to an increase in delays to operation. No unexpected safety findings were observed, and the safety profile was as expected for canakinumab and pembrolizumab.

In comparison with adjuvant treatment, systemic treatment in the neoadjuvant setting has many potential advantages, such as the early control of micrometastatic disease and the possibility of achieving tumor downstaging before operation. Furthermore, neoadjuvant treatment allows for in vivo assessment of tumor sensitivity to treatment and provides the option to administer treatment to patients before they become debilitated by operation.^24^ In addition, neoadjuvant treatment is administered in the context of a preserved tumor microenvironment, when the incorporation of immunotherapy may favor antigen exposure and immune activation.^25^ In this context, CANOPY-N was conceived to investigate whether canakinumab alone or combined with pembrolizumab would yield a significant improvement in antitumor activity compared with historical controls in the neoadjuvant setting. The results of the present study do not support this hypothesis, in line with previous studies that did not reveal a benefit for canakinumab in the adjuvant setting (as monotherapy), in the second-line metastatic setting (combined with docetaxel), and as first-line treatment (combined with platinum-based CT and pembrolizumab) for patients with NSCLC.^21^^,^^26^^,^^27^

Different reasons such as patient population and differences in end points may have contributed to the negative outcome of the abovementioned clinical trials, despite the reduction of lung cancer incidence and mortality in cardiovascular patients treated with canakinumab, as observed in CANTOS. We note that, in preclinical models, exposure to air pollution was found to promote tumorigenesis among lung cells with preexisting oncogenic driver mutations, such as *EGFR*, and IL-1β was found to be a key mediator of this process.^28^ These findings, together with those from CANTOS, raise the question of whether canakinumab can be active in the prevention of lung cancer.^18^ The Can-Prevent-Lung (NCT04789681) study is investigating whether canakinumab can induce regression of pulmonary nodules in patients at high risk of developing NSCLC, and canakinumab has demonstrated promising activity in treating patients with high-risk lung nodules in the interim analysis.^29^

Although the benefit of adding immunotherapy to neoadjuvant platinum-based CT in patients with early-stage NSCLC has been reported in randomized studies,^6^^,^^7^^,^^11^ there are still several unknowns, including the optimal duration of neoadjuvant treatment, the timing (full neoadjuvant versus perioperative), and the need for adjuvant treatment in patients submitted to neoadjuvant chemoimmunotherapy.^30^

The number of preoperative ICI cycles in neoadjuvant and perioperative, neoadjuvant plus adjuvant, trials is unclear. In the neoadjuvant ICI-CT–only approach (CheckMate 816), three cycles of preoperative ICI-CT were given.^6^ In contrast, in the perioperative ICI trials (AEGEAN, Keynote 671, and CheckMate 77T), four cycles of preoperative ICI-CT were given with no appreciable difference in pathologic complete response (24% versus 17%–25%, respectively).^10^, ^11^, ^12^ Overall, 6%–25% of patients with early-stage NSCLC are not able to complete the standard four cycles of platinum-based CT currently used in (neo)adjuvant regimens owing to toxicities.^10^, ^11^, ^12^ In the phase 2 trial of two versus three cycles of preoperative ICI-CT (neoSCORE study), there was no significant difference in pathologic complete response rates between two and three cycles.^31^ Furthermore, CT may cause long-term AEs, such as peripheral neuropathy and chronic fatigue, and therefore may compromise a patient’s quality of life with the potential to cause a delay in operation owing to toxicities.^32^, ^33^, ^34^ Therefore, a priority for future studies to improve survival outcomes for patients with NSCLC includes treatment de-escalation by shortening treatment duration or suppressing one or more of the treatment drugs.^35^ In this regard, CANOPY-N evaluated a short-course two-cycle neoadjuvant treatment regimen that has shown a favorable safety profile and allowed most patients (85.7%) to undergo operation without delay. The observation of tumor regression in some of the patients treated in this study suggests that a short-course, CT-free neoadjuvant regimen may be a viable alternative for some patients with early-stage NSCLC, although further studies are needed to identify who could benefit from this approach. In CANOPY-N, most of the patient population exhibited tumors that were PD-L1 low (1%–49%) or PD-L1 negative (<1%). Further studies will be needed to address the impact of PD-L1 expression on the necessity of adding CT and to establish whether patients with high PD-L1 (≥50%) could benefit from a CT-free immunotherapy treatment in advanced NSCLC. In addition, biomarker analyses from the CANOPY studies, including CANOPY-N, are ongoing, which may help to identify specific patients who could benefit from early anti–IL-1β treatment.^36^

Historically, the main issues with potential neoadjuvant CT treatment for patients with NSCLC include the risk of local or distant disease progression during neoadjuvant treatment, rendering the patient ineligible for the previously planned operation, and an increased intraoperative and postoperative surgical complications.^32^ Furthermore, with the recent incorporation of immunotherapy into the neoadjuvant setting, it is crucial to better characterize the impact of this regimen on patient surgical outcomes, given that scarce data on the surgical feasibility and surgical complications observed after neoadjuvant chemoimmunotherapy have been published thus far.^6^^,^^11^^,^^37^ Our findings reveal that neoadjuvant treatment with canakinumab and/or pembrolizumab was not associated with delays to operation or increased surgical morbidity compared with historical controls, suggesting that immunotherapy can be safely administered in the neoadjuvant setting.

Some limitations need to be considered when interpreting the findings from CANOPY-N. The study enrolled fewer patients than the preplanned protocol sample size, limiting the power of the efficacy analyses and preventing subgroup analyses. The study did not include an arm with the current neoadjuvant standard-of-care, platinum-based CT and immunotherapy, although this treatment was incorporated after the study was conceived.^3^ In addition, time-to-event end points were not assessed, although MPR has been found to be an adequate surrogate of survival in patients with early-stage NSCLC.^22^^,^^38^

In conclusion, canakinumab alone or combined with pembrolizumab did not reveal significant antitumor activity as neoadjuvant treatment in patients with early-stage NSCLC. However, a short-course, CT-free neoadjuvant regimen using canakinumab with or without pembrolizumab was found to be safe and did not delay operation. Intraoperative perihilar or perilobular fibrosis was rare after neoadjuvant ICI. Ongoing translational research from this study, as well as from other CANOPY studies, may help characterize the effects of IL-1β inhibition in the tumor microenvironment and identify whether any subgroup may benefit from this treatment.

## CRediT Authorship Contribution Statement

**Jay M. Lee:** Conceptualization, Investigation, Resources, Writing - original draft, Writing - review & editing, Supervision, Project administration.

**Jean-Louis Pujol:** Investigation, Writing - original draft, Writing - review & editing.

**Jun Zhang:** Conceptualization, Investigation, Resources, Writing - original draft, Writing - review & editing.

**Oleg Leonov:** Validation, Investigation, Resources, Writing - original draft, Writing - review & editing.

**Masahiro Tsuboi:** Investigation, Resources, Data curation, Writing - original draft, Writing - review & editing, Project administration.

**Edward S. Kim:** Conceptualization, Investigation, Writing - original draft, Writing - review & editing, Visualization, Supervision, Project administration.

**Calvin Ng:** Investigation, Data curation, Writing - original draft, Writing - review & editing.

**Nicolas Moreno-Mata:** Investigation, Resources, Writing - original draft, Writing - review & editing.

**Amy Cummings:** Investigation, Resources, Data curation, Writing - original draft, Writing - review & editing, Project administration.

**Ilhan Hacibekiroglu:** Investigation, Resources, Writing - original draft, Writing - review & editing.

**Abidin Sehitogullari:** Conceptualization, Methodology, Validation, Formal analysis, Investigation, Resources, Writing - original draft, Writing - review & editing, Visualization, Supervision, Project administration.

**Nirmal Veeramachaneni:** Investigation, Resources, Data curation, Writing - original draft, Writing - review & editing, Visualization.

**Cathy Spillane:** Validation, Formal analysis, Writing - original draft, Writing - review & editing, Visualization, Project administration.

**Jiawei Duan:** Methodology, Software, Validation, Formal analysis, Investigation, Data curation, Writing - original draft, Writing - review & editing, Visualization.

**Claudia Bossen:** Conceptualization, Investigation, Writing - original draft, Writing - review & editing, Supervision, Project administration.

**Alexander Savchenko:** Methodology, Validation, Writing - original draft, Writing - review & editing.

**Chiara Lobetti-Bodoni:** Conceptualization, Methodology, Validation, Formal analysis, Investigation, Writing - original draft, Writing - review & editing, Supervision, Project administration.

**Tony Mok:** Conceptualization, Methodology, Validation, Formal analysis, Investigation, Resources, Data curation, Writing - original draft, Writing - review & editing, Supervision.

**Pilar Garrido:** Investigation, Writing - original draft, Writing - review & editing, Supervision.

## Disclosure

Dr. Lee held advisory board/consultant roles for AstraZeneca, Bristol Myers Squibb, Foundation Medicine Institute, Genentech, Merck, Novartis, Regeneron Pharmaceuticals, and Roche; received research support from Bristol Myers Squibb, 10.13039/100004328Genentech, Novartis, and 10.13039/100004337Roche; reports being in the steering committee of Genentech, Merck, Novartis, and Roche; reports being in a speaker’s bureau for AstraZeneca, Bristol Myers Squibb, Genentech, and Roche; reports patents with UCLA; received consulting fees from AstraZeneca, Bristol Myers Squibb, Foundation Medicine Institute, Genentech, Merck, Novartis, Regeneron Pharmaceuticals, and Roche; received honoraria from AstraZeneca, Bristol Myers Squibb, DAVA Oncology, ecancer, Genentech, Medscape, Roche, and Targeted Oncology; and received personal fees from AstraZeneca, Bristol Myers Squibb, Foundation Medicine Institute, Genentech, Merck, Novartis, Regeneron Pharmaceuticals, and Roche. Dr. Zhang received grants/contracts from AstraZeneca, BeiGene, Genentech, Hengrui, InnoCare Pharma, Kahr Medical, Merck, Mirati, Nilogen, Janssen, and Profound Bio; received consulting fees from AstraZeneca, Novartis, Novocure, Sanofi, Takeda Oncology, Mirati, and Regeneron; received honoraria from AstraZeneca, MJH Life Sciences, Novartis, Regeneron, and Sanofi; reported being in the data safety monitoring board or advisory board of AstraZeneca, Hengrui, and Regeneron; and received research funding from Mirati. Dr. Tsuboi received grants/contracts from Merck & Co., Inc., AstraZeneca, Bristol Myers Squibb, Ono Pharmaceutical, Eli Lilly Japan, Novartis, MiRXES, National Cancer Center, and Japan Agency for Medical Research and Development; received honoraria from Johnson & Johnson, Medtronic Japan, AstraZeneca, Eli Lilly Japan, Chugai Pharmaceutical, Taiho Pharma, Bristol Myers Squibb, Ono Pharmaceutical, Novalis, Merck & Co., Inc., Daiichi Sankyo, and Amgen; reports being in the data safety monitoring board or advisory board of Chugai Pharmaceutical, AstraZeneca, Merck & Co., Inc., Novartis, and MiRXES; and reports being in the board of directors of Japan Lung Cancer Society. Dr. Ng received research funding from Medtronic Inc., Johnson & Johnson, and Noah Medical; received consulting fees from Medtronic Inc.; and received honoraria from Medtronic Inc. and Johnson & Johnson. Dr. Cummings received consulting fees from Tempus, Oncohost, and AstraZeneca. Drs. Spillane, Duan, Bossen, Savchenko, and Lobetti-Bodoni are employees and/or shareholders of Novartis. Dr. Mok received grants from AstraZeneca, Bristol Myers Squibb, G1 Therapeutics, Merck Sharp & Dohme, Merck Serono, Novartis, Pfizer, Roche, SFJ, Takeda, and XCovery; received consulting fees from AbbVie, Inc., ACEA Pharma, Adagene, Alentis Therapeutics AG, Alpha Biopharma Co., Ltd., Amgen, Amoy Diagnostics Co., Ltd., AmHeart Therapeutics, Inc., AVEO Pharmaceuticals, Inc., Bayer Healthcare Pharmaceuticals, Ltd., BeiGene, BerGenBio ASA, Berry Oncology, Boehringer Ingelheim, Blueprint Medicines Corporation, Bristol Myers Squibb, Bowtie Life Insurance Company Limited, Bridge Biotherapeutics, Inc., Covidien LP, C4 Therapeutics, Inc., Cirina, Ltd., CStone Pharmaceuticals, Curio Science, D3 Bio, Ltd., Da Volterra, Daiichi Sankyo, Eisai, Elevation Oncology, F. Hoffmann-La Roche, Ltd./ Genentech, Fishawack Facilitate, Ltd., G1 Therapeutics, Inc., GeneDecode Co., Ltd., Gilead Sciences, Inc., GLG’s Healthcare, Gritstone Oncology, Inc., Guardant Health, Hengrui Therapeutics, Inc., HiberCell, Inc., HutchMed, Ignyta, Inc., Illumina, Inc., Incyte Corporation, Inivata, IQVIA, Janssen, Lakeshore Biotech, Ltd., Lilly, Lunit USA, Inc., Loxo-Oncology, Lucence Health, Inc., Medscape LLC/ WebMD, Medtronic, Merck Serono, MSD, Mirati Therapeutics, Inc., MiRXES, MoreHealth, Novartis, Novocure GmbH, Omega Therapeutics, Inc., OrigiMed, OSE Immunotherapeutics, PeerVoice, Pfizer, PrIME Oncology, Prenetics Global Limited, Puma Biotechnology, Inc., Qiming Development (HK), Ltd., Regen Medtech Holdings Limited, Regeneron Pharmaceuticals, Inc., Roche Pharmaceuticals/ Diagnostics/ Foundation One, Sanofi-Aventis, SFJ Pharmaceutical, Ltd., Simcere of America, Inc., Synergy Research, Summit Therapeutics Sub, Inc., Takeda Pharmaceuticals HK, Ltd., Tigermed, Vertex Pharmaceuticals, Virtus Medical Group, XENCOR, Inc., and Yuhan Corporation; received honoraria from ACEA Pharma, Alpha Biopharma Co., Ltd., Amgen, Amoy Diagnostics Co., Ltd., AstraZeneca (before 1/1/19), BeiGene, BI, BMS, Daiichi Sankyo, Daz Group, Fishawack Facilitate, Ltd., InMed Medical Communication, Janssen Pharmaceutica NV, Jiahui Holdings Co. Limited, LiangYiHui Healthcare, Lilly, Lucence Health, Inc., MD Health Brazil, Medscape LLC, Merck Pharmaceuticals HK, Ltd., Merck Sharp & Dohme, MiRXES, Novartis, OrigiMed Co., Ltd., P. Permanyer SL, PeerVoice, Physicians’ Education Resource, Pfizer, PrIME Oncology, Research to Practice, Roche Pharmaceuticals/ Diagnostics/ Foundation One, Sanofi-Aventis, Shanghai BeBirds Translation & Consulting Co., Ltd., Taiho Pharmaceutical Co., Ltd., Takeda Oncology, and Touch Independent Medical Education, Ltd.; reports travel/meetings for Novartis, Roche, Pfizer, AstraZeneca, Daiichi Sankyo, BI, MiRXES, BMS, MSD, AbbVie, Zai Lab, and LiangYiHui; reports being in the data safety monitoring board or advisory board of AbbVie, Inc., ACEA Pharma, Amgen, AstraZeneca, Alentis Therapeutics AG, BerGenBio ASA, Berry Oncology, Blueprint Medicines Corporation, Boehringer Ingelheim, Bowtie Life Insurance Co., Ltd., Bristol Myers Squibb, C4 Therapeutics, Inc., Covidien LP, CStone Pharmaceuticals, Curio Science, D3 Bio, Ltd., Daiichi Sankyo, Inc., Eisai, Fishawack Facilitate, Ltd., G1 Therapeutics, Inc., Gilead Sciences, Inc., Gritstone Oncology, Inc., Guardant Health, geneDecode Co., Ltd. (uncompensated), Hengrui Therapeutics, Inc., HutchMed, Ignyta, Inc., Incyte Corporation, Imagene AI, Ltd., Inivata, IQVIA, Janssen, Lakeshore Biotech, Lily, Loxo-Oncology, Inc., Lunit, Inc., Merck Serono, Merck Sharp & Dohme, Mirati Therapeutics, Inc., MiRXES Group, Novartis, OrigiMed, Pfizer, Prenetics Global Limited, Puma Biotechnology, Inc., Roche/Genentech, Regeneron Pharmaceuticals, Inc., Sanofi-Aventis R&D, SFJ Pharmaceutical, Simcere of America, Inc., Simcere Zaiming, Inc., Takeda, Vertex Pharmaceuticals, Virtus Medical Group, XENCOR, Inc., and Yuhan Corporation; held leadership or fiduciary roles for AstraZeneca PLC, HutchMed, Aurora, and Insighta; and reports stocks with AstraZeneca, Aurora Tele-Oncology, Ltd., Biolidics, Ltd., HutchMed, Prenetics Global Limited, D3 Bio, Lunit, Bowtie Life Insurance, Lakeshore Biotech, Ltd., Loxo-oncology, Virtus Medical Group, Yinson Capital Pte., Ltd., Phanes Therapeutics, Inc., Insighta, and Alentis Therapeutics AG. Dr. Garrido reports being in the data safety monitoring board or advisory board of AbbVie, Amgen, AstraZeneca, Bayer, BMS, Boehringer Ingelheim, Daiichi Sankyo, GlaxoSmithKline, Janssen, Lilly, MSD, Novartis, Pfizer, Roche, Sanofi, and Takeda and in the steering committee of Novartis and IO Biotech IO102-Janssen. The remaining authors declare no conflict of interest.
