# Supplementary material for: CANOPY-N: A Phase 2 Study of Canakinumab or Pembrolizumab, Alone or in Combination, as Neoadjuvant Therapy in Patients With Resectable, Stage IB–IIIA NSCLC
Source: JTO Clin Res Rep. 2025 Jun 13;6(8):100859. doi: 10.1016/j.jtocrr.2025.100859 (PMC12343357; doi:10.1016/j.jtocrr.2025.100859)
Supplement: Supplementary Material [file mmc1.pdf]

Supplementary Materials

Table S1. MPR rate by central review for all treatment arms

	**Canakinumab (N = 35)**	**Canakinumab + pembrolizumab (N = 35)**	**Pembrolizumab (N = 18)**
Number of patients with MPR, n	1	6	3
MPR rate by central review (95% CI), %	2.9 (0.07–14.92)	17.1 (6.56–33.65)	16.7 (3.58–41.42)
Mean of posterior distribution of MPR rate, %	3.9	18.5	–

CI, confidence interval; MPR, major pathologic response.

Table S2. Best overall response per local investigator review

	**Canakinumab (N = 35)**	**Canakinumab + pembrolizumab (N = 35)**	**Pembrolizumab (N = 18)**
Patients with measurable disease at baseline, n (%)	35 (100)	35 (100)	18 (100)
**Best overall response**			
PR	0	3 (8.6)	2 (11.1)
SD	28 (80.0)	29 (82.9)	14 (77.8)
PD	7 (20.0)	1 (2.9)	1 (5.6)
UNK	0	2 (5.7)	1 (5.6)
ORR: CR+PR, n (%) [95% CI]	0 [0.00–10.00]	3 (8.6) [1.80–23.06]	2 (11.1) [1.38–34.71]

CI, confidence interval; CR, complete response; ORR, overall response rate; PD, progressive disease; PR, partial response; SD, stable disease; UNK, unknown.

Table S3. Adverse events, regardless of study treatment relationship, by preferred term and grade (≥10% all grade in the canakinumab arm)

	**Canakinumab (N = 35)**			**Canakinumab + pembrolizumab (N = 35)**			**Pembrolizumab (N = 18)**		
	**All grades, n (%)**	**Grade 3/4, n (%)**	**Grade 5, n (%)**	**All grades, n (%)**	**Grade 3/4, n (%)**	**Grade 5, n (%)**	**All grades, n (%)**	**Grade 3/4, n (%)**	**Grade 5, n (%)**
**Number of patients with ≥1 AE**	**31 (88.6)**	**10 (28.6)**	**3 (8.6)**	**32 (91.4)**	**9 (25.7)**	**1 (2.9)**	**15 (83.3)**	**3 (16.7)**	**1 (5.6)**
Anemia	9 (25.7)	1 (2.9)	0	1 (2.9)	0	0	1 (5.6)	0	0
Fatigue	9 (25.7)	0	0	5 (14.3)	0	0	4 (22.2)	0	0
Decreased appetite	6 (17.1)	0	0	1 (2.9)	0	0	0	0	0
Dyspnea	5 (14.3)	1 (2.9)	0	6 (17.1)	2 (5.7)	0	1 (5.6)	0	0
Bilirubin conjugated increased	4 (11.4)	0	0	1 (2.9)	0	0	1 (5.6)	0	0
Blood bilirubin increased	4 (11.4)	0	0	0	0	0	0	0	0
Cough	4 (11.4)	0	0	6 (17.1)	0	0	2 (11.1)	0	0
Lymphocyte count decreased	4 (11.4)	0	0	0	0	0	1 (5.6)	0	0
Nausea	4 (11.4)	0	0	3 (8.6)	0	0	3 (16.7)	0	0
Pneumonia	4 (11.4)	2 (5.7)	1 (2.9)	1 (2.9)	1 (2.9)	0	0	0	0

AE, adverse event.

Table S4. Overview of adverse events of special interest and imAEs

	Canakinumab (N = 35)			Canakinumab + pembrolizumab (N = 35)			Pembrolizumab (N = 18)		
	**All grades, n (%)**	**Grade 3/4, n (%)**	**Grade 5, n (%)**	**All grades, n (%)**	**Grade 3/4, n (%)**	**Grade 5, n (%)**	**All grades, n (%)**	**Grade 3/4, n (%)**	**Grade 5, n (%)**
Infections	11 (31.4)	3 (8.6)	1 (2.9)	5 (14.3)	2 (5.7)	0	7 (38.9)	1 (5.6)	1 (5.6)
Neutropenia	7 (20.0)	1 (2.9)	0	0	0	0	1 (5.6)	0	0
Abnormal liver parameters	6 (17.1)	2 (5.7)	0	6 (17.1)	2 (5.7)	0	4 (22.2)	0	0
Opportunistic infections	2 (5.7)	0	0	2 (5.7)	1 (2.9)	0	2 (11.1)	0	1 (5.6)
Pulmonary complications	1 (2.9)	1 (2.9)	0	0	0	0	0	0	0
Pulmonary hypertension and interstitial lung disease	1 (2.9)	1 (2.9)	0	0	0	0	0	0	0
Thrombocytopenia	1 (2.9)	1 (2.9)	0	0	0	0	4 (22.2)	2 (11.1)	0
imAEs	0	0	0	9 (25)	1 (2.9)	0	3 (16.7)	1 (5.6)	0
Opportunistic infections (based on SMQ narrow)	0	0	0	1 (2.9)	1 (2.9)	0	0	0	0

imAE, immune-mediated adverse event; SMQ, standardized MedDRA queries.

Table S5. imAEs by preferred term and grade

	Canakinumab (N = 35)			Canakinumab + pembrolizumab (N = 35)			Pembrolizumab (N = 18)		
	**All grades, n (%)**	**Grade 3/4, n (%)**	**Grade 5, n (%)**	**All grades, n (%)**	**Grade 3/4, n (%)**	**Grade 5, n (%)**	**All grades, n (%)**	**Grade 3/4, n (%)**	**Grade 5, n (%)**
**Number of patients with ≥1 imAE**	**0**	**0**	**0**	**9 (25.7)**	**1 (2.9)**	**0**	**3 (16.7)**	**1 (5.6)**	**0**
Hyperthyroidism	0	0	0	5 (14.3)	0	0	1 (5.6)	0	0
Hypothyroidism	0	0	0	3 (8.6)	0	0	2 (11.1)	1 (5.6)	0
Immune-mediated hepatitis	0	0	0	1 (2.9)	1 (2.9)	0	0	0	0
Rash maculo-papular	0	0	0	1 (2.9)	0	0	0	0	0
Rash pruritic	0	0	0	1 (2.9)	0	0	0	0	0

imAE, immune-mediated AE.

Table S6. Preoperative and operative surgical endpoints in CANOPY-N

Surgical and clinical endpoints	Canakinumab (N = 35)	Canakinumab + pembrolizumab (N = 35)	Pembrolizumab (N = 18)
**Preoperative**			
**Patients who had surgery, n (%)[a]**	**32 (91.4)**	**34 (97.1)**	**18 (100)**
Median time from end of treatment to surgery, weeks (min–max)	2.1 (1.6–12.3)	2.2 (1.1–20.3)	2.1 (7.0–30.0)
**Surgery within protocol-defined window, n (%)[b]**			
Yes	27 (84.4)	29 (85.3)	16 (88.9)
No	5 (15.6)	5 (14.7)	2 (11.1)
**Reason for delay to surgery**			
Site-related	3 (9.4)	0	2 (11.1)
AE-related	1 (3.1)	2 (5.9)	0
COVID-19 pandemic[c]	1 (3.1)	2 (5.9)	0
Patient decision	0	1 (2.9)	0
**Grade ≥3 preoperative TRAEs**	0	3 (8.8)	0
**Type of surgery**			
Lobectomy	14 (43.8)	24 (70.6)	15 (83.3)
Bilobectomy	4 (12.5)	4 (11.8)	0
Pneumonectomy	10 (31.3)	4 (11.8)	1 (5.6)
Wedge	2 (6.3)	0	0
Segmentectomy	1 (3.1)	1 (2.9)	0
Other	1 (3.1)	1 (2.9)	2 (11.1)
**Neoadjuvant**			
**Pretreatment clinical stage, n (%)**			
Stage IA	1 (3.1)	1 (2.9)	0
Stage IB	7 (21.9)	9 (26.5)	4 (22.2)
Stage II	21 (65.6)	21 (61.8)	12 (66.7)
Stage IIIA	3 (9.4)	3 (8.8)	2 (11.1)
**Posttreatment downstaging, n (%)**			
BOR of PR	0	3 (8.8)	2 (11.1)
Downstaging by T	NA	2 (5.9)	1 (5.6)
Downstaging by N	NA	0	0

[a]A total of four patients did not have surgery: three due to disease progression in the canakinumab arm and one due to patient decision in the canakinumab + pembrolizumab arm.

[b]The protocol-defined window for surgery was 4–6 weeks after first dose of study drug.

[c]Surgery was delayed due to COVID-19 pandemic site restrictions: two patients were delayed due to lockdown (one patient in the canakinumab + pembrolizumab arm and one patient in the canakinumab arm), and one patient in the canakinumab + pembrolizumab arm was delayed due to a COVID-19–related site issue.

AE, adverse event; BOR, best overall response; N, nodes; PR, partial response; T, tumor; TRAE, treatment-related AE.

Table S7. Proposed scales for intraoperative quantification of surgical complexity in early NSCLC after neoadjuvant immunotherapy[1]

Grade	Characteristics
**Nonmalignant lymphadenopathy**	
0	Lymphadenopathy <1 cm
1	Lymphadenopathy 1 to <2 cm
2	Lymphadenopathy 2 to <3 cm
3	Lymphadenopathy ≥3 cm
**Peripheral (pleural) fibrosis**	
0	No fibrosis
1	Mild fibrosis (no substantial impact on conduct of surgical resection)
2	Moderate fibrosis (requires increased effort and dissection during resection but otherwise does not severely impact the conduct of the surgery)
3	Severe fibrosis (substantially impacts the conduct of the operation by increasing the duration of or blood loss during the surgery, or requires converting minimally invasive to open surgery)
4	Severe fibrosis resulting in unresectability
**Central vs. peripheral lung cancer**	
1	Central (inner two-thirds of lung)
2	Peripheral (outer one-third of lung)
**Perihilar/lobar or mediastinal adhesions**	
0	No fibrosis
1	Mild fibrosis (no substantial impact on conduct of surgical resection)
2	Moderate fibrosis (requires increased effort and dissection during resection but otherwise does not severely impact the conduct of the surgery)
3	Severe fibrosis (substantially impacts the conduct of the operation by increasing the duration of or blood loss during the surgery, or requires converting minimally invasive to open surgery)
4	Severe fibrosis resulting in unresectability

1. Lee JM, Kim AW, Marjanski T, et al: Important Surgical and Clinical End Points in Neoadjuvant Immunotherapy Trials in Resectable NSCLC. *JTO Clin Res Rep* 2:100221, 2021

Statistical analysis

The best overall response (BOR) was determined from response assessments undertaken on or before end-of-treatment (EOT). Only tumor assessments performed before the start of any further antineoplastic therapies were considered in the assessment of BOR. A new anticancer therapy was defined as any systemic secondary anticancer therapy, or any radiotherapy received during or post-study treatment. Overall response rate (ORR) and two-sided Clopper–Pearson exact binomial 95% confidence interval are presented by treatment arm.

Surgical feasibility rate was defined by the percentage of patients who underwent surgery. Both the feasibility rate and two-sided exact binomial 95% confidence interval are presented by treatment arm.
